# A Spotlight on the Role of Radiomics and Machine-Learning Applications in the Management of Intracranial Meningiomas: A New Perspective in Neuro-Oncology: A Review

**DOI:** 10.3390/life12040586

**Published:** 2022-04-14

**Authors:** Lara Brunasso, Gianluca Ferini, Lapo Bonosi, Roberta Costanzo, Sofia Musso, Umberto E. Benigno, Rosa M. Gerardi, Giuseppe R. Giammalva, Federica Paolini, Giuseppe E. Umana, Francesca Graziano, Gianluca Scalia, Carmelo L. Sturiale, Rina Di Bonaventura, Domenico G. Iacopino, Rosario Maugeri

**Affiliations:** 1 Neurosurgical Clinic AOUP “Paolo Giaccone”, Post Graduate Residency Program in Neurologic Surgery, Department of Biomedicine Neurosciences and Advanced Diagnostics, School of Medicine, University of Palermo, 90127 Palermo, Italy; lapo.bonosi@gmail.com (L.B.); robertacostanzo3@gmail.com (R.C.); sofiamusso.sm@gmail.com (S.M.); umberto.emanuele.benigno@gmail.com (U.E.B.); rosamariagerardimd@gmail.com (R.M.G.); robertogiammalva@live.it (G.R.G.); federicapaolini94@gmail.com (F.P.); gerardo.iacopino@gmail.com (D.G.I.); rosario.maugeri1977@gmail.com (R.M.); 2 Department of Radiation Oncology, REM Radioterapia SRL, 95125 Catania, Italy; gianluca.ferini@grupposamed.com; 3 Gamma Knife Center, Trauma Center, Department of Neurosurgery, Cannizzaro Hospital, 95100 Catania, Italy; umana.nch@gmail.com; 4 Unit of Neurosurgery, Garibaldi Hospital, 95124 Catania, Italy; fragraziano9@gmail.com (F.G.); gianluca.scalia@outlook.it (G.S.); 5 Division of Neurosurgery, Fondazione Policlinico Universitario A. Gemelli IRCCS, Università Cattolica del Sacro Cuore, 00100 Rome, Italy; carmelo.sturiale@policlinicogemelli.it (C.L.S.); rina.di.bonaventura@hotmail.it (R.D.B.)

**Keywords:** radiomics, machine learning, deep learning, meningioma, medical imaging, neuro-oncology

## Abstract

**Background**: In recent decades, the application of machine learning technologies to medical imaging has opened up new perspectives in neuro-oncology, in the so-called radiomics field. Radiomics offer new insight into glioma, aiding in clinical decision-making and patients’ prognosis evaluation. Although meningiomas represent the most common primary CNS tumor and the majority of them are benign and slow-growing tumors, a minor part of them show a more aggressive behavior with an increased proliferation rate and a tendency to recur. Therefore, their treatment may represent a challenge. **Methods:** According to PRISMA guidelines, a systematic literature review was performed. We included selected articles (meta-analysis, review, retrospective study, and case–control study) concerning the application of radiomics method in the preoperative diagnostic and prognostic algorithm, and planning for intracranial meningiomas. We also analyzed the contribution of radiomics in differentiating meningiomas from other CNS tumors with similar radiological features. **Results:** In the first research stage, 273 papers were identified. After a careful screening according to inclusion/exclusion criteria, 39 articles were included in this systematic review. **Conclusions:** Several preoperative features have been identified to increase preoperative intracranial meningioma assessment for guiding decision-making processes. The development of valid and reliable non-invasive diagnostic and prognostic modalities could have a significant clinical impact on meningioma treatment.

## 1. Introduction

In recent years, thanks to the ability of computers to mimic problem-solving capabilities of the human mind, a rise in the number of studies concerning the efficacy of artificial intelligence (AI) as an aid tool throughout the diagnostic pathway in neuro-oncology has been recorded [1,2,3]. Machine learning (ML) and deep learning (DL) represent two subfields of AI, and brain radiomic analysis represents one of their applications to traditional medical imaging. Radiomics allows extraction of quantitative - and ideally reproducible - information called *radiomic features* about tissue and lesion characteristics from diagnostic images. The peculiarity of this method is that medical images contain and reveal more information of disease-specific processes than the human eye can see, thus “unveiling the invisible” [4,5]. Radiomics exploits mathematical extraction of the spatial distribution of signal intensities and pixel inter-relationships to quantify disease-specific information, exceeding the human subjective interpretation of radiological imaging. Some examples of these features are related to heterogeneity and shape, and to their potential changes over time on serial imaging, and several studies demonstrated how the degree of tumor heterogeneity could represent an additional prognostic element determinant on treatment choice or survival [6]. Radiomic features could, alone or in combination with other tumor developing extrapolated data (genomic, transcriptomic, or proteomic data) or with demographic and histologic data, help in the diagnostic and treatment tumor challenges, and in patient stratification [7,8].

Meningiomas are the most common primary tumors of the central nervous system (CNS), showing an increasing incidence over the past decade owing to population aging and improvements in the diagnostic capacity [9,10,11]. Histological criteria still guide intracranial meningioma treatment and, together with surgical resection, influence patients’ prognosis, even with the latest revised WHO classification (fifth edition, 2021) [12]. The majority of diagnosed meningiomas are benign, slow-growing tumors that can be effectively treated by complete surgical excision. Atypical or anaplastic meningiomas (grade II and III, respectively, according to the previous WHO 2016 grade) account for, respectively, <15% and <5% of them, and show more aggressive biological behavior, growth, recurrence tendency, and mortality rates. Early diagnosis and accurate recognition of higher malignancy meningiomas are crucially important to evaluate prognostic outcomes and guide treatment; this is the basis on which more advanced non-invasive MRI techniques (such as diffusion or perfusion sequences) have been previously employed [13], and where currently ML algorithms can show their promising potential to extract fundamental information on grading or on the risk of recurrence [14]. The applications of radiomics and radiogenomics (the study derived from the integration of radiographical and genomic data) have already achieved important proven results in glioma tumors, and some studies indicate increasing diagnostic, grading, and prognostic capabilities [15,16], corroborating its potential role in improving the diagnosis and patients’ prognosis.

The aim of this paper is to provide a perspective of the current data pertaining to the evolving progress of the radiomics method in meningioma tumors for both diagnosis and prognosis, and to identify potential tumor features which may affect medical and surgical decision-making, and treatment outcomes. We give a panoramic view of meningioma radiomic features, and their impact on medical daily life identifying limitations and gaps, as well as their accuracy, and promising future directions in radiomics endpoints and radiologically challenging aspects of meningioma interpretation. Lastly, our review tries to expand on the search parameters in distinguishing differential diagnosis.

## 2. Methods

### 2.1. Study Design

**Figure 1:**
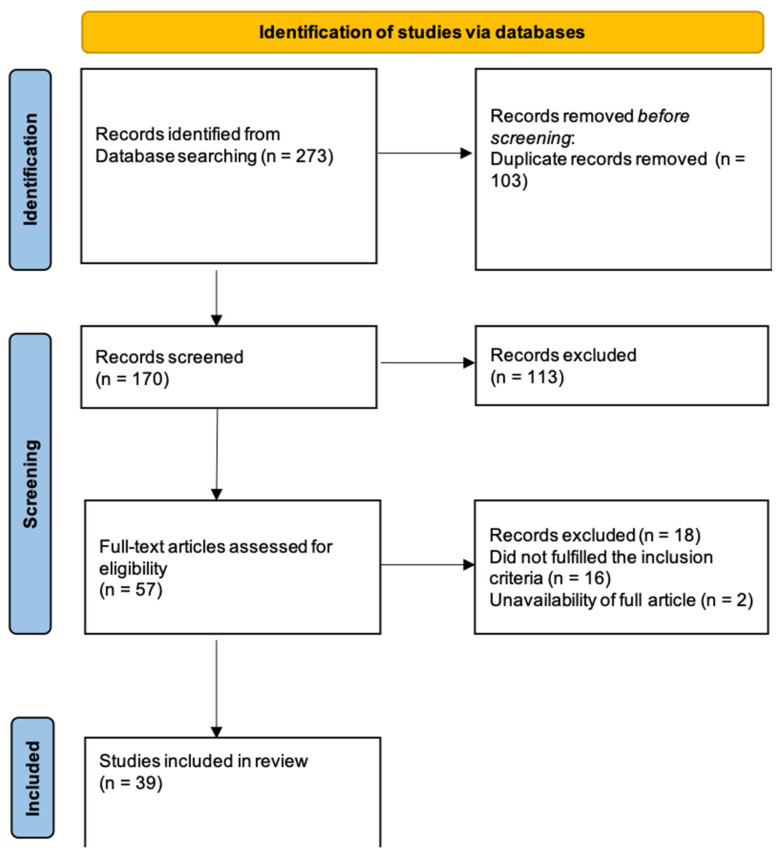
Flow-diagram of the results of this systematic review according to PRISMA statement.

According to PRISMA (Preferred Reporting Items for Systematic Reviews and Meta-Analyses) guidelines, an extensive systematic literature review was manually performed on PubMed, MEDLINE, and Scopus databases by two independent authors (S.M., and L.B.). A combination of the following medical subject headings (MeSH) and free text terms were used: “radiomics”, “machine learning”, “deep learning”, “artificial intelligence” AND “meningioma”, and “intracranial meningioma”, with no limits in terms of date of publication until January 2022. Details of the search strategy are shown in Figure 1.

### 2.2. Eligibility Criteria

Meta-analyses, retrospective studies, and review were included. Non-English works, studies regarding CNS tumors other than meningioma, and studies lacking full text were excluded. Each article was screened according to the topic of this review and only those discussing the application of radiomics in brain meningiomas were selected.

### 2.3. Data Extraction

Among the selected articles, we included those concerning meningioma and other CNS tumors which share several radiological features, and those describing how radiomics could help in the differential diagnosis between radiological features. The available data are shown in Supplementary Materials Table S1, and included authors, year, study design, study objective, MRI and radiomics acquisition features, and some relevant additional notes.

## 3. Results

**Figure 2:**
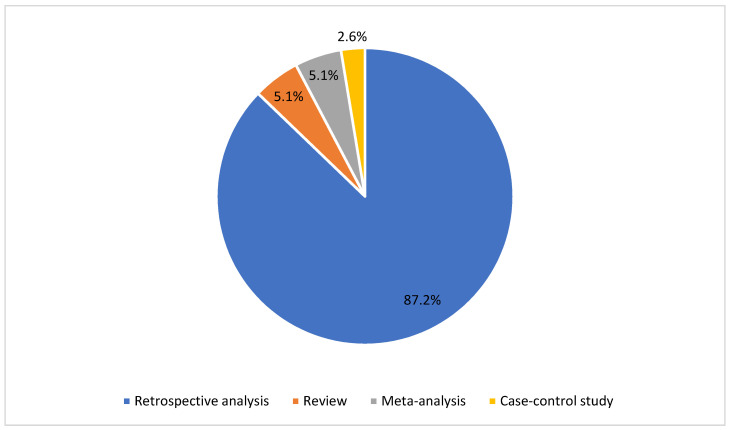
Pie chart of the radiomics studies included in this systematic review according to the type of article.

**Figure 3:**
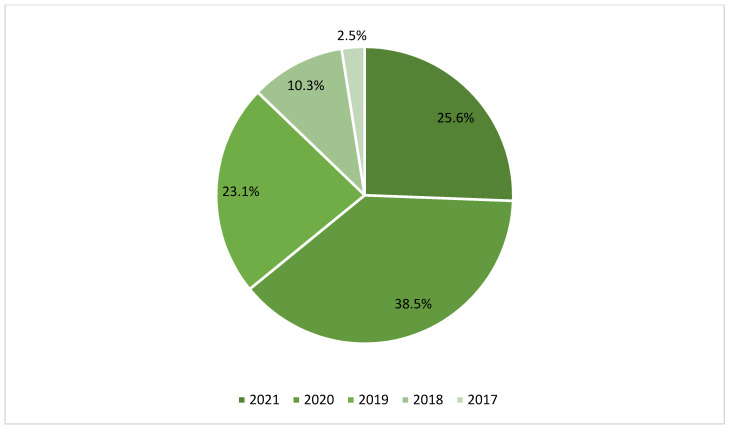
Pie chart of the radiomics studies included in this systematic review according to the year of publication.

Through a careful analysis of the literature, 273 papers were identified. These records were screened according to the aforementioned inclusion/exclusion criteria. Thus, 103 duplicates were removed and 113 were later excluded because they were not consistent with the topic of this review and our inclusion criteria. From the 57 full-text articles assessed for eligibility, 16 further articles were excluded because of 1) the lack of relevance about radiomics in meningioma tumors, and 2) the lack of full text. As a result, 39 articles were included in this systematic review. Interobserver agreement was calculated by Cohen’s Kappa Statistic, and κ was 0.89. In Supplementary Materials Tables S1 and S2, and Figure 2 and Figure 3, the main characteristics of the included studies are summarized; 2 of them are meta-analysis, 2 review, 1 case–control study, 34 retrospective study (and 1 of them in a multicenter evaluation). The journal type consisted of 21 clinical journals (53.8%), 17 imaging journals (43.6%), and 1 informatic journal (2.6%).

## 4. Discussion

The current limitations in medical imaging techniques provide an opportunity for developing more advanced sub-visual feature analysis, and to handle demanding expectations in tumor treatment. In this context, AI methodologies have been exponentially used and radiomics represents a promising approach in individualized oncological management as a new low-cost tool [17]. One of the most relevant advantages is that radiomics analysis can reveal the heterogeneity within a region through identifying different sub-regions, thus representing the spatial complexity of a disease [8,17]; in fact, while biopsies represent only a small portion of a tumor, and usually at just a single anatomic site, radiomics can capture heterogeneity across the entire tumor volume [18]. The key element in the radiomics process is to define the region in which radiomic features are studied, called the region of interest (ROI) in two-dimensional (2D) or the volume of interest (VOI) in three-dimensional (3D). Image segmentation is the first step in the radiomics process, and can be performed manually, semi-automatically, or fully automatically, the latter using DL algorithms. DL-based image segmentation reduces some of the considerable intra- and interobserver variability of the manual and semi-automated processes, as well as procedure time; nonetheless, this method is inferior in terms of accuracy compared to the others. Next steps in radiomic process are image processing, feature extraction, and feature selection/dimension reduction [4,17,19]. Image processing is the phase where images from which radiomics features will be extracted are homogenized (with respect to pixel spacing, grey-level intensities, and bins of the grey-level histogram); several studies have shown that different imaging acquisition parameters can influence the nature of derived radiomics features [7,8]. The dimension reduction is a multi-step process that works, through the application of filters, to exclude non-reproducible, redundant, and non-relevant features from the dataset; as a consequence, this step is essential for generating valid and reliable results, for building statistical and ML models, and for enhancing the prediction accuracy of the method. Over the past decade, there was a substantial growth in radiomics research for brain tumors, and the following text is a window into the most pertinent literature about the application of radiomics models in meningiomas.

### 4.1. Preoperative Meningioma Grading

Most studies about radiomics focused on the possibility of applying ML approaches to the preoperative prediction of meningiomas grading, with important repercussions on the choice of therapeutic strategy. Preoperative imaging has a central role for the assessment of CNS tumors, and MRI is the gold standard for its high soft-tissue resolution and no radiation; however, it is not straightforward enough to distinguish different meningioma subtypes from conventional MRI sequences, even the most advanced ones, and DL analysis has gradually achieved excellent results. Previous studies illustrated that conventional MRI images, especially including contrast-enhanced (CE)-T1 and FLAIR, were capable of differentiating between high- and low-grade meningiomas, through the analysis of shape, size, location, tumor-brain interface, necrosis, and heterogeneous tumor enhancement [20]. The observers’ knowledge and experience influenced the evaluation of most of the radiomics features, and this may have limited consequent clinical benefit. Functional MRI techniques, such as diffusion weighted imaging (DWI) and susceptibility weighted imaging (SWI), have also been reported to facilitate tumor grade stratification. Several studies based on DWI demonstrated that high-grade meningiomas tend to present with lower apparent diffusion coefficient (ADC) values than low-grade entities, but the results remained controversial. By providing information on tumor vasculature, intra-tumoral calcification, and microhemorrhages, SWI contributes to distinguishing the histological grade of glioma tumors; however, only a few studies have explored the potential value of SWI in predicting the histological grade of meningiomas [20].

CE may influence radiomics acquisition regarding the true grayscale, uniformity, texture depth, and depth thickness, and non-enhanced MRI sequences usually provide a better reflection of the pathological changes [21].

The working group of Han et al. [21] demonstrated that radiomics features extracted from FLAIR MRI sequences and analyzed with an SVM classifier provide the best results in predicting the histopathological grade of meningiomas, and were superior to the well-established DWI analyses. The use of other MRI sequences in radiomics process has been explored by other authors. Balzano et al. [22] showed that the application of DL on ADC maps contributes to a high diagnostic accuracy in discriminating between benign and atypical/anaplastic meningiomas (according to the old WHO 2016 CNS tumors classification), whereas the same application on CE-T1WI gives inaccurate results, consistent with what was previously reported. In the authors’ opinion, a heterogeneous enhancement is generally associated with grade II or III meningiomas but not exclusively, because several grade I lesions may show a cystic and heterogeneous appearance (the grade is referred to in the old WHO 2016 classification); thus, the wide variability in CE-T1WI features could limit the classification performance of DL analysis. The use of ADC and SWI radiomics models was still demonstrated by other studies [20,22,23,24]. Focusing on the characteristic of grade II meningiomas (according to the old WHO 2016 CNS tumors classification), Kalasauskas et al. [25] underlined that the presence of a majority cystic component and the presence of high cluster prominence are the semantic and radiomic characteristics most associated with a high relapse rate [26].

Interestingly, in some recent works the possibility of using radiomics to differentiate meningiomas of specific histotypes was explored. Park et al. [23] showed how, using various texture parameters extracted from T1, CE-T1, and DTI MRI sequences, it is possible to preoperatively determine various histotypes related to meningiomas, in particular by differentiating fibroblastic from non-fibroblastic forms. Fibroblastic meningioma subtypes have been discovered to show a firmer mass consistency, thus requiring hard-working dissection, and influencing preoperative treatment strategy and presumptive surgical outcome. Niu’s working group [27] demonstrated how, based on radiomic features extracted from CE-T1WI, it is possible to differentiate various histological subtypes among meningiomas (in particular between three histotypes: meningothelial, fibrous, and transitional) with excellent sensitivity and specificity, stressing how imaging-based radiomics could significantly improve diagnostic performance and provide new therapeutic strategies in highly selected patients.

In a recent systematic review and meta-analysis by Ugga et al. [28], the authors addressed the problem of methodological quality of retrospective studies published about radiomic analyses in meningiomas. Radiomics demonstrated great results in representing a promising valid option in the preoperative evaluation of tumor grading and improvement of management of meningiomas. Nonetheless, the authors especially emphasize the current limitations linked to the applicability of these methods in a clinical setting, due to low average radiomics quality score (RQS—6.96, 19%) of the articles included in their systematic review, updated to 2021, and reflecting a lacking overall methodological quality and high-quality results among the large number of publications in this field.

### 4.2. Preoperative Prediction of Ki-67 in Benign Meningioma

Another interesting field of application of radiomics in meningioma is related to the preoperative ability to correlate the expression of Ki-67 to Grade I meningiomas (according to the old WHO 2016 CNS tumors classification). Meningioma grade I is the most diagnosed in clinical practice, and this histological grade is characterized by a heterogeneous clinical presentation, rate of growth, and risk of recurrence. Several studies showed that higher Ki-67 in tumor biopsy analysis correlates with increased risk of recurrence, and acts as a predictor of recurrence. The working group of Khanna [29] described that their model combining sequences from DWI and CE-T1WI and morphologic features (increased peritumoral edema shape eccentricity and enhancing tumor extent) can be used to differentiate WHO grade I meningiomas based on Ki-67 expression with good accuracy. A recent study, in fact, outlined that the extent of resection is an independent risk of recurrence in grade I meningiomas when Ki-67 expression is more than 4.5%. In fact, gross total resection (GTR) and supra-total resection (STR) show similar risk of recurrence [30]. This reinforces the concept that the ability to preoperatively presume meningiomas behavior could guide the best tailored therapeutic strategy and also the time frame for imaging follow-up.

### 4.3. Prediction of Brain Invasion as an Indirect Tool for Recurrence and Poor Prognosis

Meningioma brain invasion was documented to be independently associated with risks of tumor progression, recurrence, and a poor prognosis, and it became a stand-alone criterion for differentiation of grade II in the old 2016 WHO classification of CNS tumors, apart from the presence of histopathologic criteria of atypia, with consequent prognostic implications. Thus, it was assumed that the use of brain invasion as a non-invasive imaging biomarker for predicting meningiomas with higher grades of malignancy could be employed for enhancing clinical decision-making. Previous studies investigated brain invasion through a qualitative radiological approach, considering loss of the CSF cleft sign, cortical penetration, irregular shape, and presence/absence of edema. Otherwise, radiomics permits a quantitative and objective approach of tumor infiltration using the brain-to-tumor interface and the analysis of intensity distributions, spatial relationships, and texture heterogeneity, and provides information on the disruption of the pial surface. Joo et al. [31] constructed a combined model of the top six radiomics features from the brain-to-tumor interface on T2WI and CE-T1WI plus the volume of peritumoral edema which showed better prediction of brain invasion, and marked improved diagnostic value over the volume-only edema model. Similar results were shared by other working groups [32,33,34,35], underlining the great potential of radiomics in this particular subfield and the treatment strategy implication that all this entails. The invasion of bone adjacent to the meningioma represents another crucial preoperative aspect that can be evaluated using a radiomics approach and can contribute to devising surgical strategies. It has been shown that using features selected from CE-T1 and T2 MRI images it was possible to predict the risk of bone invasion with good accuracy [36].

### 4.4. Prediction of Meningioma Mass Consistency

The possibility of predicting the consistency of the meningioma mass from preoperative MRI is another important key-point on which the authors focused [37]. Tumor consistency represents one of the factors (together with tumor size, the presence/absence of an arachnoid plane of separation between tumor and adjacent structures, and tumor vascularization) that could influence surgical removal difficulty, length of operation time, degree of resection, postoperative complications, and tumor recurrence [38]. In fact, the removal of soft tumors can result easier with shorter surgical time, greater extent of resection, and less bleeding, while firm consistency meningioma are more challenge, particularly at the skull base. In this context, an approach based on the analysis of radiomic features could give key information and guide the most advantageous individualized operation schemes, as demonstrated by some recent studies [39,40]. Cepeda et al. [39] analyzed the consistency of meningiomas by combining the intraoperative elasticity measured through the ultrasound elastography (IOUS-E) as a reference parameter with the analysis of the preoperative radiomic features from MRIs. The best radiomic features selected were the conventional kurtosis in CE-T1, the gray-level zone length matrix of the ADC, and the conventional first-quartile of T2WI. Magnetic resonance elastography (MRE) is an available non-invasive technique to quantitatively and preoperatively determine tumor stiffness, but the lack of availability in most centers together with insufficient spatial resolution, and the difficulty in evaluating highly vascular tumors, still limit its use [40,41]. 

### 4.5. Differential Diagnosis between Meningioma and Other CNS Tumors

Noteworthy is the possibility of using quantitative data extracted from MRI to make a differential diagnosis between meningiomas and other tumor histotypes who shared some MRI characteristics. Maki et al. [42], demonstrated how, using an algorithm of DL based on convolutional neural networks (CNNs) and radiomic features extracted first from T2 WI and CE-T1WI, it is possible to differentiate a spinal meningioma from a schwannoma with excellent specificity and sensitivity. In the past, it was assumed that intracranial solitary fibrous tumor was a subtype of meningioma. While angiomatous meningioma is a rare WHO 2016 grade I histological subtype of meningioma with a good prognosis that can be effectively cured through resection, solitary fibrous tumor is a more aggressive type of neoplasm, which can relapse and metastasize to extracranial tissues. Therefore, preoperative identification of both is essential. Dong et al. [43] and Li et al. [44] both showed how it was possible to differentiate a solitary fibrous tumor from an angiomatous meningioma with high accuracy, and excellent sensitivity and specificity based on data extracted from T1WI, CE-T1WI, and T2WI. Similar results, although exploiting different sequences including CE-T1WI, FLAIR, and DWI, were also obtained by Wei’s working group [45].

### 4.6. Prognostic Implications

If applied to a clinical setting, radiomics could represent a key tool paving the way for ultra-personalized medicine. The ability to evaluate tumor characteristics, otherwise met only after tissue biopsy, could revolutionize the management of meningioma patients leading to the creation of increasingly accurate and reliable prognostic models. As a matter of fact, many efforts were made to create prognostic models based on radiomics, clinical and demographic characteristics that were able to predict outcomes, local failure (LF), and overall survival (OS) [46]. Based on supervised learning models associated with algorithms for regression and classification, the possibility of predicting both the LF and the OS with good accuracy has been demonstrated [47]. Zhang et al. [48], demonstrated how, using images extracted from T1-C, T2, and DWI sequences, it is possible to predict with high accuracy (overall prediction accuracy 90%) the risk of tumor recurrence and/or progression. In this context, a fundamental role is played by the implementation of the extraction, segmentation, and processing systems, as well as by the ML algorithms. Many studies have shown how the use of various radiomic features and different types of data analysis algorithms are associated with quite heterogeneous results in accuracy, with potential consequences on the development of reliable diagnostic and prognostic systems [49,50,51].

### 4.7. Limitations and Future Perspectives

Radiomics is certainly an evolving procedure that is rapidly showing promising results in neuro-oncology for its diagnostic and prognostic implications, as assessed for gliomas. Nevertheless, it faces several difficulties and currently remains confined to the scientific literature for meningiomas management. What appears immediately clear to readers is data sparsity. While scarcity can be otherwise predictable for its recent spread (Figure 3), sparsity becomes a relevant feeling in the readers’ mind when they face the multitude of variations in acquisition, interpretation, software and MRI application, and more (Supplementary Materials Table S1), and try to understand what the technique itself is. Due to the lack of standardization methods, of sufficient reports, and of limited open-source data available, the reproducibility of radiomic results is faulty, and the risk of false-positive results prevent its use in routine clinical practice [52]. Alongside this, the interpretability of radiomics features is also questionable, and the lack of comparison with well-established prognostic and predictive factors can mislead (e.g., causation vs. correlation) in routine clinical decision process. Patients’ characteristics, tumor geometry, and quality of imaging acquisition can also influence the levels of noise and the presence of artifacts. Classification accuracy in meningiomas can be challenging due to the surrounding edema, the hyperintensity deriving from dural attachment or vascular branches, which can modify tumor boundaries and influence detection and segmentation processes [53]. Radiomics studies found in this systematic review are mainly retrospective without standardized guidelines or control of imaging protocols, and with low levels of evidence; thus, further prospective studies are needed to corroborate the value of radiomics in meningiomas. Compared to gliomas, these studies are fewer, making it difficult to perform an accurate comparative study. Three different radiomics guidelines were defined addressing critical aspects and quality of radiomics studies, trying to reduce or prevent all the previously mentioned issues [54]: the Radiomics Quality Score (RQS), the Transparent Reporting of a multivariable prediction model for Individual Prognosis or Diagnosis (TRIPOD) [55], and the Image Biomarker Standardization Initiative (IBSI). This further check can aid in achieving reproducibility of radiomic studies, allowing easier clinical translation and comparison of radiomics features for meningioma patients at different institutions [56], and should be implemented and spread in ongoing and prospective meningioma radiomics studies. At present, fully automated extraction of radiomic features is supposedly the direction of pertinent future research. Taking this route, radiomics could also represent a weapon in incidental meningioma management without histological diagnosis, helping in easier and more individualized clinical decision-making [22,54]. Finally, considering all the issues discussed above, radiomics is actually of restricted use in routine clinical practice. Protocols of MRI acquisition and elaboration, and software algorithm use need to be established, and to allow for comparison and to homogenize the results interpretation everywhere. Significant advances in glioma characterization and classification are now following the path of radiogenomics; in a future workflow, considering the spreading literature research about immunological environments in meningiomas and the aforementioned relevant acquisition in glioma management with the introduction of radiogenomics, could radiomics’ features shift together with other biological, genetical, and immunological aspects to create a more integrated approach to meningioma, such as *radioimmunogenomics*?

## 5. Conclusions

Radiomics could represent not just an additional supportive tool and an automation of the diagnostic process, but a source of key tumor information that, overcoming conventional imaging parameters and together with additional biological data (clinical, genetic, molecular, histopathological, etc.), could have a great potential in the clinical and surgical decision-making process. It would be advisable for the future of neuro-oncology to employ advanced resources, such as radiomics and radiogenomics, in meningioma management in order to provide less invasive and tumor-specific diagnosis, characterization, and subsequently more personalized treatment strategies with greater precision, and to ultimately optimize prognosis and patient care. No conclusive results are yet known about how these preoperative radiomics analyses may impact patients’ outcomes; thus, more standardized and reproducible methods of data interpretation, available databases, and prospective large-scale multi-institutional clinical trials are needed.

## Supplementary Materials

The following supporting information can be downloaded at: https://www.mdpi.com/article/10.3390/life12040586/s1: Table S1: Summary of the main characteristic of the articles included in this systematic review; Table S2: Literature review search overview, search string doc.
