# Supplementary material for: A Spotlight on the Role of Radiomics and Machine-Learning Applications in the Management of Intracranial Meningiomas: A New Perspective in Neuro-Oncology: A Review
Source: Life (Basel). 2022 Apr 14;12(4):586. doi: 10.3390/life12040586 (PMC9026541; doi:10.3390/life12040586)
Supplement: Supplementary file 1 [file life-12-00586-s001.zip › Search String.pdf]

(((radiomics) OR (machine learning) OR (deep learning) OR (artificial intelligence) AND (meningioma)) OR ((radiomics) OR (machine learning) OR (deep learning) OR (artificial intelligence) AND (intracranial meningioma)))
