# Supplementary material for: A Spotlight on the Role of Radiomics and Machine-Learning Applications in the Management of Intracranial Meningiomas: A New Perspective in Neuro-Oncology: A Review
Source: Life (Basel). 2022 Apr 14;12(4):586. doi: 10.3390/life12040586 (PMC9026541; doi:10.3390/life12040586)
Supplement: Supplementary file 1 [file life-12-00586-s001.zip › Table S1.pdf]

Table S1. Summary of the main characteristic of the articles included in this systematic review.

	Author, year	*No. of patients/studies*	*Type of study*	*Objective*	*MRI sequences*	*Radiomic analysis description*	*Results*	*Additional information*
1	***Ugga et al, 2021***	23 studies included in the systematic review, 8 for the meta-analysis	Meta-analysis	To evaluate the methodological quality of studies using radiomics for diagnostic and predictive purposes	-	ICC to evaluate inter-reader reproducibility and methodological quality assessment. Quality Assessment of Diagnostic Accuracy Studies tool was used to assess methodological quality analysis and risk of bias.	Moderato to good inter-reader reproducibility was demonstrated (ICC=0.75, 95% confidence intervals). Overall AUC of 0.88 (95%CI=0.84-0.93) for the meta-analysis with a standard error of 0.02.	-
2	***Won et al, 2021***	25 studies included	Meta-analysis	To evaluate the quality of radiomics studies using a RQS, TRIPOD, and IBSI.	-	-	The quality of radiomics studies for meningioma is insufficient.	RQS, TRIPOD, IBSI.
3	***Li et al, 2021***	284 patients	Retrospective study	To construct predictive model for brain invasion in WHO grade II meningioma from preop MRI	T1, T2 and CE-T1	Manual segmentation was conducted to extract the tumor region. Semi-automatic segmentation was used to acquire the tumor-to-brain interface region.	Integrated nomogram were constructed and CSNR showed the best performance in predicting brain invasion (AUC values were 0.905 in the training set and 0.895 in the test set).	SPSS 22.0, R, Python 4.0, and Deepwise DXAI Platform for statistical validation, analysis, and visualization.
4	***Zhai et al, 2021***	172 patients	Retrospective study	To construct predictive model for preop meningioma consistency	CE-T1, T2, FLAIR, ADC	Manual segmentation for the ROI (using 3D-Slicer). 28 features were selected (PyRadiomics package) to construct the radiomics nomogram. 5 supervised ML algorithms were applied (RF, KNN, SVM, LR, Ada).	The nomogram showed a good sensitivity and specificity with AUCs of 0.861 and 0.960 in train and test cohorts, respectively. LR classifier had the highest prediction efficacy.	Python (v3.7.6) and R software (v4.0.0) for statistical analysis.
5	***Ko et al, 2021***	128 patients	Retrospective study	To preop predict progression/recurrence of meningioma	T1, T2, FLAIR, GRE T2, DWI, CE-T1	FCM clustering-based algorithm for tumor segmentation. FLIRT for combining the ROIs. SVM with Gaussian kernel for texture feature extraction. For comparison with the radiomics model in the prediction of P/R in meningiomas, ADC values (b = 1,000 s/mm2) on DWI were measured manually.	Bone invasion, ADC value, and SVM score were high-risk factors for P/R ($p < 0.05$) with hazard ratios of 7.31, 4.67, and 8.13, respectively. Using the SVM score, an AUC of 0.80 with optimal cutoff value of 0.224 was obtained for predicting P/R. Patients with higher SVM scores were associated with shorter progression-free survival ($p = 0.003$).	SPSS (V.24.0, IBM, Chicago, IL, USA) for statistical analysis. Chi-square (or Fisher's exact test) and Mann–Whitney U tests for the evaluation of clinical parameters and conventional MRI findings.
6	***Xiao et al, 2021***	505 patients	Retrospective and multicenter study	To evaluate comparative performance of	T1, FLAIR, CE-T1	Manual segmentation process on CE-T1. PyRadiomics was	The most predictive radiomics features are almost entirely	R software for Statistical analyses.

				radiomics signatures derived from varying regions related to intratumoral region (whole tumor), peritumoral region (brain-tumor interface), and the amalgamation of the two regions (combine region) in predicting brain invasion.		used to select radiomics features.	generated from GLCM and GLDM statistics. The addition of PEV to radiomics features enhanced model discrimination of invasive meningiomas.	
7	***Khanna et al, 2021***	306 patients	Retrospective study	To use a ML algorithm to predict Ki-67 in WHO grade I meningiomas	T1, EC-T1, T2, FLAIR ADC, DWI	Image preprocessing was performed using CaPTk v.1.8.1. Images were segmented using ITK-SNAP v3.8.0. Feature extraction was performed in CaPTk over the segmentation labels. The model was based on the extracted radiomic features in the discovery cohort by the LASSO feature selection method, wrapped with linear SVM classification through a nested CV approach.	An AUC of 0.84 (95% CI: 0.78-0.90) with a sensitivity of 84.1% and specificity of 73.3% was achieved in the discovery cohort. When applied to the replication cohort, a similar high performance with an AUC of 0.83 (95% CI: 0.73-0.94), sensitivity and specificity of 82.6% and 85.5%, respectively.	Unpaired 2-tailed t-test, Chi-square, and Fisher's exact tests, and IBM SPSS v 24.0 for statistical analyses.
8	***Cepeda et al, 2021***	18 patients	Retrospective study	To developed a high-precision classification model capable of predicting consistency of meningiomas based on the radiomic features in preop MRI	T2, CE-T1, ADC	Automatic segmentation of the whole tumor volume using ITK-SNAP software. Open-source software LifEx version 6.0 for texture analysis. Open-source software Orange version 3.26 for feature selection. Five possible scoring methods (Information Gain, Gini index, ReliefF, and FCBF). A classification system was built using 6 different algorithms (a logistic regression with LASSO, Naive Bayes, k-nearest neighbors, RF, SVM, and a Multi-layer Perceptron algorithm.	The best-ranked radiomic features were obtained from CE-T1, EDC map, and T2 WI. The combination of Information Gain and ReliefF filters with the Naive Bayes algorithm resulted in an AUC of 0.961 and classification accuracy of 94%.	Shapiro-Wilk test for the distribution of the continuous variables. A univariate analysis was performed using the Fisher exact test for categorical variables. Student t test and the Mann-Whitney U test for statistical analyses.
9	***Xiao et al, 2021***	136 patients	Retrospective study	To evaluate the value of 3D radiomics features in predicting postop CEE in a primary cohort and a validation cohort	CET1 and T2WI	IYK-SNAP software for 3D ROI reconstructions; PyRadiomics for 4 quantitative radiomics feature: shape and size, first-order, textural and wavelet features; Wilcoxon rank sum test, elastic	Clin-radiomics model combining peritumoral edema and tumor size, and radiomics signature with an AUC of 0.91 in the primary cohort and 0.83 in the validation cohort.	

						net and RFE algorithm to reduce redundant features.		
10	***Zhang et al, 2021***	5088 patients	Retrospective study	To design a DL algorithm and evaluate the performance in detecting meningioma lesions and grade classification	CE-T1	Automated segmentation classification based on the ResNet model (using a connection method called "shortcut connection"). The segmentation model included PSPNet and CRF.	In all patients, the PSPNet successfully detected the presence of meningiomas, with accuracy of 100%. Manual segmentation and automated DL-based segmentation correlated well with tumor volume.	Student's t test or the Mann–Whitney U test with MATLAB version 2018b software for statistical analysis.
11	***Laukamp et al, 2020***	126 patients	Retrospective study	To establish a dedicated meningioma DL model based on routine MRI data and to evaluate its performance for automated tumor segmentation.	T1, T2, CE-T1, FLAIR	IntelliSpace Discovery for manual segmentations (assisted/semi-automated). Established tools were used for MRI image preprocessing (SPM8, Wellcome Trust Centre for Neuroimaging; Intellispace Discovery, Philips Healthcare).	The comparison of the DL model and manual segmentations revealed average dice coefficients of 0.91± 0.08 for CE tumor volume and 0.82± 0.12 for total lesion volume. DL-based automated segmentation yielded high segmentation accuracy, comparable to manual inter-reader variability.	The JMP software for statistical analyses. Wilcoxon signed rank test was determination of statistical difference.
12	***Joo et al, 2020***	604 patients	Retrospective study	To develop and validate an imaging-based model combining interface radiomics and peritumoral edema to predict brain invasion by meningioma	T2, EC-T1	Semi-automated algorithm for defining ROI for the brain-to-tumor interface. Three radiomics feature groups of first-order features (n = 17), texture features (n = 162), and wavelet-transformed features (n = 1432) were extracted with an automated process running in Matlab (R2016a, Mathworks).	The combined model showed the highest performance for prediction of brain invasion in the training (AUC 0.97; 95% CI: 0.95–0.98) and validation sets (AUC 0.91; 95% CI: 0.84–0.98), and improved diagnostic performance over volume of peritumoral edema only (AUC 0.76; 95% CI: 0.66–0.86).	The Wald test was applied to calculate P-values for each parameter.
13	***Han et al, 2020***	131 patients	Retrospective study	To develop a radiomics model to predict the histopathological grading of meningiomas	T1 FLAIR, T2, CE-T1 FLAIR	All VOI files were uploaded to the Radcloud platform. The Variance Threshold method and Select K Best method were used to preprocess features. Six machine learning classifiers were used in this study: LR, RF, KNN, DT, SVM, and XGBoost.	The best performance of the radiomics model for the degree of differentiation was obtained by SVM (AUC 0.956; 95% confidence interval (CI), 0.83–1.00; sensitivity, 0.87; specificity, 0.92; f1-score, 0.90).	The Spearman method was used to calculate the redundancy between feature parameters.
14	***Neromyliotis et al, 2020***	-	Review	To highlight recent studies about feasibility of radiomics in meningioma surgery	-	-	The field remains at an early stage of research	-

15	***Hu et al, 2020***	514 patients	Retrospective study	To investigate the prediction performance of radiomic models based on multiparametric MRI in predicting the meningioma grade	T1, T2, CE-T1, DWI	Semiautomatic method. Open-source python package Pyradiomics 2.2.0 for radiomic feature extraction. LOOCV method to estimate the classification performance of different radiomic models. LASSO feature selection method to search the optimal radiomic features before the classification experiments.	The cMRI + ADC + SWI model demonstrated the best performance without or with subsampling, which AUCs were 0.84 and 0.81, respectively.	R version 3.3.2 software, t student test and Chi-square test for statistical analyses. Data visualization was completed with R packages: ggplot2, pROC, corrplot and complex Heatmap.
16	***Maki et al, 2020***	82 patients	Retrospective study	To evaluate the performance of CNN in differentiating between spinal schwannoma and meningioma	T2, CE- T1	Python programming language, version 3.6.7 and Google's open source DL framework Tensorflow, version 1.12.0 were used to construct the CNN architecture.	The AUC of ROC curves of the CNN based on T2WI and contrast-enhanced T1WI were 0.876 and 0.870, respectively.	SAS and JMP for statistical analyses. Youden index for sensitivity, specificity, and accuracy.
17	***Gu et al, 2020***	-	Review	To compare different performances of popular algorithms and to discuss several possible future directions in the development of radiomic applications in meningiomas.	-	-	Radiomics analysis for meningiomas is a promising new area of research based on the development of computational advances. The current correlation is mainly between the imaging phenotypes and meningioma grades.	-
18	***Chu et al, 2020***	126 patients	Retrospective study	To explore the application value of MRI radiomics based on enhanced-T1WI in the prediction of meningiomas grade	CE-T1	Semiautomatic segmentation for ROI reconstruction. The segmented images were imported into the AK (Artificial Intelligence Kit V3.0.0.R, GE Healthcare) software for texture features extraction.	9 feature parameters with more significant predictive value were selected (including 3 histogram features, 1 morphological feature, 1 Haralick feature, 2 GLCM features, 1 RLM feature, and 1 gray-level size zone matrix feature). The AUC in the training group and the test group were 0.958 and 0.948, respectively.	R software for statistical analysis. The "glmmet" software package in R software was used for the LASSO calculation. "stats" package was performed for Logistic regression establishing a prediction. model.
19	***Dong et al, 2020***	97 patients	Retrospective study	To differentiate between Intracranial solitary fibrous tumor/hemangiopericytoma by using radiomics	T1, T2, CE-T1	Manual segmentation for ROI reconstruction. The segmented images were imported into the AK software for texture features extraction.	Four models were constructed resulting in an AUC of 0.885 (sensitivity 76.1%, specificity 87.9%) for T1WI model, 0.918 (73.1%, 95.5%) for T2WI model, 0.815 (55.2%, 93.9%) for	Modeling was performed using the language R.

							contrasted T1WI model, and 0.959 (92.5%, 84.8%) for the combined sequence model.	
20	***Kandemirli et al, 2020***	108 patients	Retrospective study	To determine the diagnostic utility of first-order texture parameters in differentiating meningiomas with and without brain invasion	T1, CE-T1	Segmentation was only performed on CE-T1 WI. Tumor margins were manually delineated using the "polygon mode". TexRAD uses a filtration-histogram method applies a LoG filter to remove image noise. For texture analysis, TexRAD was used.	In detection of brain invasion, random forest model based on whole tumor segmentation had an AUC of 0.988 (95 % CI 0.976–1.00) with a cross validated value of 0.74 (95 % CI 0.45-0.96). For differentiation of grade I from grade II meningiomas with brain invasion, the AUC was 0.999 (95 % CI 0.995–1.00) and 0.81 (95 % CI 0.61-0.99) in the training and validation cohorts, respectively. Similarly, when using only the single largest slice, the cross-validated AUC to distinguish BI versus NBI and BI versus grade I meningiomas was 0.67 (95 % CI 0.47, 0.92 and 0.78 (95 % CI 0.52, 0.95) respectively.	Model fitting, tuning and crossvalidated computation of AUC was performed using the MachineShop package in R version 4.0.2.
21	***Zhang et al, 2020***	490 patients	Retrospective study	To predict bone invasion in meningiomas using radiomic signatures based on preoperative, CE-T1 and T2-weighted MRIs.	T1, T2, CE-T1	For each patient, 1227 radiomic features were extracted from CE-T1 and T2, respectively. Spearman's correlation and LASSO regression analyses were performed to select the most informative features.	8 radiomic features were selected from CE-T1 and T2 respectively, and 3 models were built using radiomic features. The radiomic models derived from CE-T1 alone or a combination of CE-T1 and T2 had the best performance in predicting risk of bone invasion, with AUCs in the training dataset of 0.714 [95% CI, 0.660-0.768] and 0.722 [95 % CI, 0.668-0.776] and in the test datasets of 0.715 [95 % CI, 0.632-0.798]	5-fold cross-validation was used to compare the performance of different classification algorithms, and logistic regression was chosen to predict the risk of bone invasion.

							and 0.713 [95 % CI, 0.628-0.798], respectively.	
22	***Kalasauskas et al, 2020***	76 patients	Retrospective study	To assess the value of radiomic for the prediction of tumor recurrence.	FLAIR, CE-T1, ADC		Cystic component was significantly associated with tumor recurrence OR 21.7, 95% CI 3.8–124.5) and shorter progression-free survival (33.2 vs. 80.7 months, p < 0.001). Radiomic features showed no predictive value in univariate analysis, while multivariate analysis demonstrated significant predictive value of high cluster prominence HR 5.89 (1.03–33.73) and cystic component (HR 20.21 (2.46–166.02)) for tumor recurrence	SPSS software, Version 26.0 (IBM Corporation) for univariate and multivariate regression analysis as well as ROC curve analysis.
23	***Morin et al, 2020***	303 patients	Retrospective study	To investigate prognostic models based on clinical, radiologic, and radiomic feature to preoperatively identify meningiomas at risk for poor outcomes.	T1, T2, FLAIR, DWI, CE-3D SPGR T1	Manual segmentation using commercially available software (MIM Software Inc.) Feature extraction using a scale of 3 mm and a quantization level of 32 fixed bins. For incorporation into prognostic models, the initially large set of radiomic features was reduced to fewer than 15 features using a supervised false-positive avoidance methodology to reduce feature correlation and dimensionality.	Multivariate analysis revealed that apparent diffusion coefficient hypointensity (HR 5.56, 95% CI 2.01–16.7, P = .002) was associated with high grade meningioma, and low sphericity was associated both with increased LF (HR 2.0, 95% CI 1.1–3.5, P = .02) and worse OS (HR 2.94, 95% CI 1.47–5.56, P = .002). Both radiologic and radiomic predictors of adverse meningioma outcomes were significantly associated with molecular markers of aggressive meningioma biology (somatic mutation burden, DNA methylation status, and FOXM1 expression). Integrated prognostic models combining clinical, radiologic, and radiomic features demonstrated improved accuracy	R software for statistical analysis.

							for meningioma grade, LF, and OS (area under the curve 0.78, 0.75, and 0.78, respectively) compared to models based on clinical features alone.	
24	***Zhang et al, 2020***	1728 patients	Case-control study	To predict the risk of brain invasion in meningioma by incorporating radiomic and clinical features.	CE-T1, T2	Manual segmentation with an open-source ITK-SNAP software. The pyradiomic platform to extract standardized radiomic features. The LASSO regression with three-fold cross-validation was used to select the radiomic features highly correlated with brain invasion.	Sixteen features were significantly correlated with brain invasion. The clinic-radiomic model derived from the fusing MRI sequences and sex resulted in the best discrimination ability for risk prediction of brain invasion, with AUCs of 0,857 (95% CI, 0,831-0,887) and 0,819 (95% CI, 0,775-0,863) and sensitivities of 72,8% and 90,1% in the training and validation cohorts, respectively.	Python 3.7.1, R software and IBM SPSS 22.0 for Windows for statistical analysis.
25	***Wei et al, 2020***	292 patients	Retrospective study	To distinguish between Intracranial hemangiopericytoma (IHPC) and meningioma by using radiomics.	T1, CE-T1, T2	Pyradiomics tool for radiomic features extraction from segmented tumor. Feature selection was primarily conducted by assessing feature stability and reproducibility via calculating the CCC and the ICC. The AIC was used to select optimal incorporated factors and utilized logistic regression modeling to perform HMDT construction.	The HMDT displayed remarkable diagnostic ability, with AUCs of 0.985 and 0.917 in the training and validation cohorts, respectively.	Mann–Whitney U-test was applied to select diagnosis outcome-related radiomic features with a $p < 0.05$.
26	***Laukamp et al, 2019***	211 patients	Retrospective study	To apply radiomics-based shape and texture analysis on routine multiparametric MRI for meningioma grading.	T1, T2, FLAIR, DWI, ADC, CE-T1	PyRadiomics-package used to generate 990 shape/texture features. Step-wise dimension reduction and robust radiomics feature selection were performed.	4 statistically independent radiomics features were identified as showing the strongest predictive values for higher tumor grades: roundness-of-FLAIR-shape (AUC: 0.80), cluster-shades-of-FLAIR/CE-T1-grey-level (AUC: 0.80), DWI/ADC-grey-level-variability (AUC: 0.72), and FLAIR/CE-T1-grey-level-energy (AUC: 0.76). The combination of the	R software for statistical analysis. Simple and multiple logistic regression analyses using the Akaike Information Criterion. Ten-fold cross validation to determine the misclassification rate of these models.

							features led to an AUC of 0.91 for the differentiation of grade I and grade II meningiomas.	
27	***Ke et al, 2019***	184 patients	Retrospective study	To evaluate the feasibility of differentiation between benign and nonbenign meningiomas by using radiomics	T1, T2, CE-T1	Manual segmentation for ROI reconstruction. A piecewise affine histogram-based method25 was implemented to normalize the image intensities, reduce brightness and contrast variations, and minimize the effect of inter-scanner differences. 4 categories of texture feature (GLCM, GLRLM, GLSZM, NGTDM) were extracted. The SVM classifier was used to construct the models.	The multiparametric MRI model demonstrated the best performance for differentiating between benign and nonbenign meningiomas in both the training and external validation cohorts (AUC 0.91, Acc 89%, F1 0.88, Sen 0.93, and Spec 0.87 in the training cohort; AUC 0.83, Acc 80%, F1 0.77, Sen 0.84, and Spec 0.78 in the validation cohort).	ANOVA for continuous variables, and chi-squared test or Fisher's exact test for categorical variables.
28	***Chen et al, 2019***	150 patients	Retrospective study	To investigate whether texture analysis-based machine learning algorithms could help devise a non-invasive imaging biomarker for accurate classification of meningiomas using machine learning algorithms.	CE-T1	Texture analysis was conducted with LIFEx software. Texture features included features from histogram-based matrix and shape-based matrix from the first order and features from GLCM, GLZLM, NGLDM, GLRLM from second or higher order. Classification models were built with different combinations of 3 selection methods (distance correlation, LASSO, and GBDT) and 2 multiclass algorithms (LDA and SVM). The diagnostic performances of models were evaluated with confusion matrix based on which the AUCs, accuracy, and Kappa value of models were calculated.	Confusion matrix showed that the LDA-based models represented better diagnostic performances than SVM-based models. The highest accuracy among LDA-based models was 75.6%, shown in the combination of Lasso + LDA. The op timal models for SVM-based models was Lasso + SVM, with accuracy of 59.0% in the testing group. One of the SVM-based models, GBDT + SVM, was overfitting, suggesting that this model was not suitable for application.	-
29	***Zhang et al, 2019***	60 patients	Retrospective study	To implement MRI radiomics to predict progression/recurrence in skull base meningiomas.	T2, ADC, CE-T1	GLCM texture features and 13 histogram-based parameters were calculated from the tumor ROI. RF algorithms were utilized via Bootstrap-aggregated decision trees to evaluate the	The 3 most significant parameters included in the final radiomics model were T1 max probability, T1 cluster shade, and ADC correlation. In the radiomics model, the accuracy for	Statistical package SPSS for statistical analysis.

						importance of these features.	prediction of P/R was 90%; by comparison, the accuracy was 83% using ADC values measured from manually placed tumor ROI.	
30	***Zhu et al, 2019***	181 patients	Retrospective study	To non-invasively differentiate meningioma grades by DLR model based on routine post-contrast MRI.	T1, T2, CE-T1	DLR features referred to the outputs of convolutional neural networks' hidden layers (2048 deep learning features were extracted).<br>For feature extraction, the Xception tool box applied in Keras was used. Random forest algorithm was used to calculate the feature importance Classification was performed with LDA method.	The DLR signature comprised 39 deep learning features and showed good discrimination performance in both the primary and validation cohorts. AUC, sensitivity, and specificity for predicting meningioma grades were 0.811(95% CI, 0.635–0.986), 0.769, and 0.898 respectively in the validation cohort. DLR performance was superior over the hand-crafted features. Calibration curves of DLR model showed good agreements between the prediction probability and the observed outcome of high-grade meningioma.	Python software (version 3.6) for statistical analysis.
31	***Li et al, 2019***	67 patients	Retrospective study	To assess whether a ML model based on TA could differentiate malignant haemangiopericytoma from angiomatous meningioma.	CE-T1, FLAIR, DWI	The extracted features included grey-level histogram, cooccurrence matrix, run-length matrix, autoregressive model and wavelet transform.	Malignant HPCs were found to have larger sizes, slighter degrees of peritumoral edema compared with AMs ($P < 0.05$), and more serpentine-like vessels. The AUC of the enhanced T1WI-based classifier was 0.90, significantly higher than that of T2-FLAIR-based or DWI-based classifiers (0.77 and 0.73)	R software for statistical analysis. Chi2 test and Fisher's exact test for the significance and correlations of the non-continuous features. The continuous data among clinical and texture features were presented as the mean ± SD and compared using T test. AUC values from the ROC curves were compared using a DeLong test.
32	***Banzato et al, 2019***	117 patients	Retrospective study	To determine the diagnostic accuracy of a DCNN in the	CE-T1, ADC	Inception-V3 and Alexnet deep neural networks pretrained	Application of the Inception-V3 DCNN on ADC maps	Diagnostic accuracy of the DCNN was

				differentiation of the histopathological grading of meningiomas from MRI.		on a large-scale database were used for image classification.	provided the best diagnostic accuracy results, with an AUC of 0.94 (95% CI, 0.88–0.98). Application of AlexNet on ADC maps had a low discriminating accuracy, with an AUC of 0.68 (95% CI, 0.59–0.76) and a high misclassification rate. Discriminating accuracy of both DCNNs on CE-T1 images was low, with Inception-V3 displaying an AUC of 0.68 (95% CI, 0.59–0.76) and AlexNet displaying an AUC of 0.55 (95% CI, 0.45–0.64).	tested using a leave-one-out cross-validation procedure. ROC and AUC were used to asses DCNN performance.
33	***Hamerla et al, 2019***	138 patients	Retrospective study	To test the hypothesis that a ML approach can distinguish grade 1 from higher gradings in meningioma patients using radiomics features.	T2,CE-T1, subtraction maps, FLAIR, ADC	Four machine learning classifiers were scored on combinations of MRI modalities: RF, XGBoost, SVM and MLP. Extraction of radiomics features was carried out using the previously reported PyRadiomics library implemented in Python.	The best AUC of 0.97 (1.0 and 0.97 for sensitivity and specificity) was observed for the combination of ADC, ADC of the peritumoral edema, T1, CE-T1, Sub and FLAIR-derived features using only 16 of the 10,914 possible features and XGBoost.	-
34	***Niu et al, 2019***	241 patients	Retrospective study	To investigate the differential diagnostic value of radiomics features on meningioma.	T1, CE-T1, T2, FLAIR, DWI	AI Kit software for radiomics analysis was used. A total of 385 features were extracted from CE T1-WI. Then the 385 features were divided into 5 categories: histogram parameters, texture parameters, form factor parameters, GLCM parameters, and RLM parameters.	Differentiation accuracies of the Fisher discriminant anal ysis model for each group were 99.4%, 98.8%, 100% and 100%, respectively; leave one out cross validation method was achieved for each group with the accuracies of 91.3%, 95.0%, 100%, and 94.2%, respectively.	Statistical analyses were performed using IBM SPSS. Fisher discrimination analysis and LOSSA for the model validation.
35	***Park et al, 2018***	136 patients	Retrospective study	To evaluate the role of radiomics features of CE-T1, ADC, and FA maps, in the differentiation of grades and histological subtypes of meningiomas.	T1, CE-T1, DTI	In total, 90 texture features and eight morphology features were calculated. The 3 categories of texture features included histogram-based, co-occurrence matrix-based, and run-length matrix-based parameters. For feature subset	The best classification system for the prediction of meningioma grades had an AUC of 0.86 (95% CI, 0.74–0.98). The accuracy, sensitivity, and specificity of the best classifier were 89.7, 75.0, and 93.5%, respectively.	-

						selection, either no subset selection or RFE was performed. These feature selection methods were combined with SVM and RF ML methods. Low-frequency intensity nonuniformity removed by applying the N4 bias correction algorithm. CE-T1 image intensities were normalized using WhiteStripe packages.		
36	***Lu et al, 2018***	152 patients	Retrospective study	To assess whether TA based on ADC maps could be used for meningioma classification.	T1, T2, FLAIR, ADC, DWI	The process of feature selection was completed by MaZda and R software. The process of classification was performed using R studio.	The ADC value alone was unable to distinguish three WHO grades of meningiomas. The ML classifiers based on clinical, morphological features and ADC value could achieve equivalent diagnostic performance (accuracy = 62.96%) compared to two experienced neuro-radiologists (accuracy = 61.11% and 62.04%).	R software for statistical analysis. ANOVA and the Friedman test for valuing significance and correlations of the continuous features of the different groups.
37	***Laukamp et al, 2018***	56 preop MRI dataset	Retrospective study	To investigate DLM performance in automated detection and segmentation of meningiomas in comparison to manual segmentation.	T1,T2, CE-T1, FLAIR	DLM based on the DeepMedic architecture using a deep 3D convolutional neural network, followed by a 3D fully connected network to remove false positives.	Automated segmentations correlated strongly with manual segmentations: average Dice coefficients were 0.81 ± 0.10 (range, 0.46-0.93) for the total tumour volume (union of tumour volume in FLAIR and CE-T1) and 0.78 ± 0.19 (range, 0.27-0.95) for contrast-enhancing tumor volume in CE-T1.	JMP Software for statistical analyses.
38	***Hale et al, 2018***	128 patients	Retrospective study	To compare the performance of ML algorithms to standard statistical methods when predicting meningioma grade.	T2	6 preoperative imaging and demographic variables were included: tumor volume, degree of peritumoral edema, presence of necrosis, tumor location, patient sex, and presence of a draining vein to construct the models. Tumor volume was calculated using manual tracing.	The artificial neural networks outperformed all other ML models across the true-positive versus false-positive (receiver operating characteristic) space (AUC = 0.8895).	MATLAB R2016b for mathematical analysis. All models were subjected to hyperparameter optimization individually, and cross-validation was performed. Chi-square statistics for logistic

								regression curves.
39	***Coroller et al, 2017***	175 patients	Retrospective study	To predict meningioma grade by semantic and radiomic features.	CE-T13D MPRAGE or SPGR	10 binary semantic features were scored. Radiomic features (total of 1,055) were extracted from images using a custom Matlab script. Classifications were made using the RF method.	12 radiographic features (8 radiomic and 4 semantic) were significantly associated with meningioma grade, which exibit necrosis/hemorrhage (ORsem = 6.6, AUCrad = 0.62–0.68), intratumoral heterogeneity (ORsem = 7.9, AUCrad = 0.65), non- spherical shape (AUCrad = 0.61), and larger volumes (AUCrad = 0.69) compared to low-grade tumors. Radiomic and semantic classifiers could significantly predict meningioma grade (AUCsem = 0.76 and AUCrad = 0.78). Combining them increased the classification power (AUCradio = 0.86).	Five models for grade classification based on: clinical, location, semantic, radiomic, radiographic (combined radiomic and semantic features), and a combined model integrating all features above.
